# Quantitative method for the assignment of hinge and shear mechanism in protein domain movements

**DOI:** 10.1093/bioinformatics/btu506

**Published:** 2014-07-30

**Authors:** Daniel Taylor, Gavin Cawley, Steven Hayward

**Affiliations:** D’Arcy Thompson Centre for Computational Biology, School of Computing Sciences, University of East Anglia, Norwich, NR4 7TJ, UK

## Abstract

**Motivation:** A popular method for classification of protein domain movements apportions them into two main types: those with a ‘hinge’ mechanism and those with a ‘shear’ mechanism. The intuitive assignment of domain movements to these classes has limited the number of domain movements that can be classified in this way. Furthermore, whether intended or not, the term ‘shear’ is often interpreted to mean a relative translation of the domains.

**Results:** Numbers of occurrences of four different types of residue contact changes between domains were optimally combined by logistic regression using the training set of domain movements intuitively classified as hinge and shear to produce a predictor for hinge and shear. This predictor was applied to give a 10-fold increase in the number of examples over the number previously available with a high degree of precision. It is shown that overall a relative translation of domains is rare, and that there is no difference between hinge and shear mechanisms in this respect. However, the shear set contains significantly more examples of domains having a relative twisting movement than the hinge set. The angle of rotation is also shown to be a good discriminator between the two mechanisms.

**Availability and implementation:** Results are free to browse at http://www.cmp.uea.ac.uk/dyndom/interface/.

**Contact:** sjh@cmp.uea.ac.uk.

**Supplementary information:** Supplementary data are available at *Bioinformatics* online.

## 1 INTRODUCTION

Multi-domain proteins can be regarded as comprising quasi-globular regions connected by linkers that allow their relative movement. Consequently, domain movements are often engaged in protein function in a wide variety of contexts, including catalysis, transport, signaling and immune response (Bennet and Huber, 1984; Gerstein *et al.*, 1994; Schulz, 1991). In many of these cases, domain movements occur on the binding of a ligand. For example, in multi-domain enzymes, the binding of the substrate in the interdomain cleft causes the domains to close trapping the substrate in the specific environment necessary for catalysis. Well-known examples include citrate synthase (Wiegand and Remington, 1986), liver alcohol dehydrogenase (Eklund *et al.*, 1981) and F1-ATPase β subunit (Abrahams *et al.*, 1994).

Experimentally determined information on protein domain movements at the atomic level comes from the structures of proteins in different states solved primarily by X-ray crystallography and nuclear magnetic resonance spectroscopy. These different states may relate to function when they are within the functional cycle, but they may also be due to differences in the experimental conditions under which the structures were solved, or could be due to natural or engineered mutations. These structures, deposited in the Protein Data Bank (PDB) (Berman *et al.*, 2000), are a rich source of information on protein domain movements. Thus, multiple structures of proteins have been used to analyse and classify domain movements in a number of studies over the past 20 years (Amemiya *et al.*, 2011; Brylinski and Skolnick, 2008; Gerstein *et al.*, 1994; Hayward, 1999; Qi and Hayward, 2009; Sinha *et al.*, 2001; Taylor *et al.*, 2013).

**Fig. 1.**
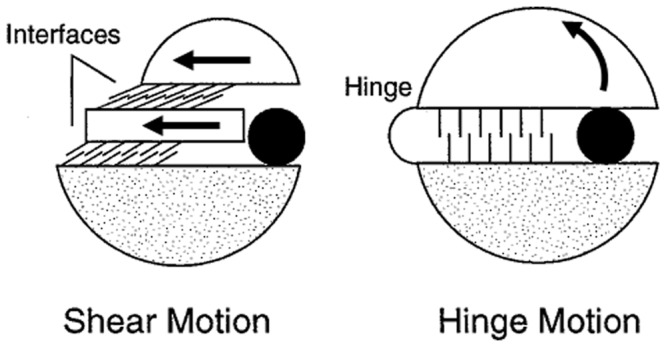
Shear and hinge mechanisms. Based on the depiction given in Figure 1 in Gerstein *et al.* (1994) illustrating the shear and hinge mechanisms. The arrows indicate the direction of movement from the closed (depicted) to the open conformation

The concepts of hinge and shear mechanisms in domain movements were first described by Gerstein *et al.* (1994) in their influential review article. Subsequently, the DataBase of Macromolecular Movements (DBMM) appeared online with further examples (Gerstein and Krebs, 1998). Hinge motions were described as those where the domains approach each other perpendicular to the plane of the interface. Shear movements, in contrast, have a preserved domain interface where the domains have a relative movement along the plane of the interface. Hinge movements would allow for large relative movement of the domains, whereas shear movements would be limited by the preserved side-chain packing at the interface. Although few details were given, it seems that these assignments were made intuitively, probably using molecular graphics software to compare the open and closed structures. This approach obviously limits the number of cases that can be classified in this way, and is also open to criticism in that it is not reproducible. Despite these limitations, the fact remains that, for some proteins, domain closure occurs through a simple ‘pacman’ opening-closing movement, whereas for others the movement is more complex with the two domains remaining in contact during the domain movement. To investigate this further, one would need to develop an automatic method for assigning hinge and shear that uses quantitative and reproducible methods. With this method, one would be able to classify a much larger number of domain movements allowing the further investigation of these two types of mechanisms. To do this, quantities are required that capture the essential difference between hinge and shear movements. The descriptions used in the articles that describe the hinge and shear movements point to two alternative approaches: one based on the relationship between the domain interface and the movement, the other based on residue contact changes (e.g. via ‘interdigitating sidechains’, or newly established contacts, see Fig. 1). In this article, we have taken the latter approach.

In our previous work (Taylor *et al.*, 2013), changes in interdomain residue contacts that occur in the domain movement were used to define four types of elemental contact changes: maintained, exchanged partner, exchanged pair and new. A maintained contact change is where the same pair of residues is found to be in contact in both conformations. An exchanged-partner contact change is one where the same residue is found to be in contact with two different residues in the two conformations, as would occur in a sliding movement. An exchanged-pair contact change is one where the residue contact pair in one conformation and the residue contact pair in the other conformation have no residues in common, as would occur in a see-saw movement. A new contact change is one where there is a contact pair in one conformation but no contact pair in the other conformation and might occur in an open to closed domain movement. Counting the number of instances of each elemental contact-change type is non-trivial, but a solution was found by the use of so-called ‘dynamic contact graphs’ (Taylor *et al.*, 2013). If a domain movement is predominantly shear, one would expect it to have a relatively large number of either maintained or exchanged-partner contact changes, whereas if a domain movement is predominantly hinge, then one would expect it to have a relatively large number of exchanged-pair or new contact changes.

Here machine learning is used, which uses the number of instances of each of these four types of contact changes for each domain movement to ‘learn’ from the DBMM to make hinge and shear assignments optimally. The movements in a much larger dataset can then be assigned to hinge and shear categories automatically. In a sense, this approach has allowed us to extract some essence of the subjective approach used to assign hinge and shear movements in the DBMM so that these assignments can be made to a larger dataset.

The language, and the figure used in the review article by Gerstein *et al.* (1994) to depict the shear movement, appears to have led to an interpretation of a shear movement to mean a relative translational movement of the domains, i.e. there is little or no rotational movement involved. Figure 1 illustrates hinge and shear movements based on the figure and descriptions given in the review article (Gerstein *et al.*, 1994). A similar figure has appeared in a review article on protein flexibility and drug design (Teague, 2003).

One might wonder why it is important to make a distinction between a rotational motion and a translational motion in the context of protein domain motions. The key point is that rotations will be locally controlled at specific hinge sites, whereas a translational motion would not be controlled at specific sites. Sites where control over a functional movement is exercised are potential target sites for therapeutic molecules. For example, a drug molecule binding to a single hinge site in an enzyme might prevent domain closure and subsequent catalysis of the natural substrate occurring just as effectively as an inhibitor that binds to the active site. The assignment of a domain movement as occurring via a translation would seem to preclude it from this form of alternative drug-site targeting.

## 2 MATERIALS AND METHODS

The basic data are the 2035 unique domain movements from the non-redundant database of protein domain movements, NRDPDM (Qi *et al.*, 2005). The domain movements were determined by the DynDom program (Hayward and Berendsen, 1998; Hayward and Lee, 2002). These unique movements come from 1578 families, which means that some domain movements are from the same family. Individual cases from this dataset are available to browse at http://www.cmp.uea.ac.uk/dyndom. To simplify the analysis, only those cases with two domains were used. Of the 2035 cases, 1822 are two-domain proteins. This dataset will be referred to as ‘NRDPDM2d’.

DBMM (Gerstein and Krebs, 1998) is available online (http://www.molmovdb.org) and has 37 examples of domain motions classified as ‘predominantly shear’ and 75 examples of domain motions classified as ‘predominantly hinge’.

### 2.1 Residue contact definition

Contact between residue i and residue j means any heavy atom of residue i is within 4 Å of any heavy atom of residue j. However, before the set of pair-wise contacts between residues in each domain and for each conformation is determined, residues at the boundaries of the domains assigned by DynDom as bending regions were removed, as were residues close to the interdomain screw axis (any heavy atom of the residue within 5.5 Å of the axis). The reason for this is that they would be expected to have maintained contacts irrespective of the nature of the domain movement.

### 2.2 Counting the number of elemental contact changes in a domain movement

**Fig. 2.**
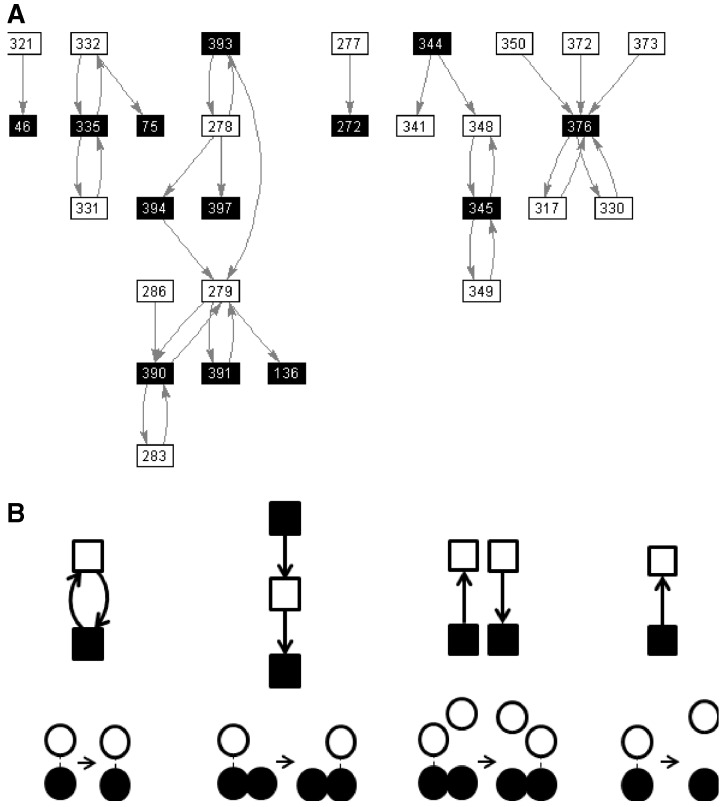
DCG and decomposition. (**A**) The DCG for the domain movement between conformation 1 (PDB accession code: 1CTS) and conformation 2 (PDB accession code: 1CSH) in citrate synthase. A filled square corresponds to a residue in domain A, and an open square corresponds to a residue in domain B with the residue number written in the square. An arrow from a residue in A to a residue in B indicates a contact between the residues in conformation 1. An arrow from a residue in B to a residue in A indicates a contact between the residues in conformation 2. (**B**) The elemental DCGs for ‘maintained’, ‘exchanged-partner’, ‘exchanged-pair’ and ‘new’ that represent the pairwise residue contact changes depicted underneath each graph. The DCG in (A) is decomposed into these elemental DCGs to give N_maint_ = 10, N_exchpart_ = 2, N_exchpair_ = 2 and N_new_ = 6. The prediction value for this domain movement is 0.55, which puts it in the Mixed class

Let {(a_1i_,b_1i_)}, i = 1, N_1_ be the set of ordered pairs of residue numbers corresponding to residues, a_1i_ from domain A, and b_1i_ from domain B, making a contact in conformation 1. Let {(a_2i_, b_2i_)}, i = 1, N_2_ be the equivalent set for conformation 2. From these two sets, a ‘dynamic contact graph’ (DCG) can be created as described by Taylor *et al.* (2013). A DCG is a directed graph, an example of which from citrate synthase is shown in Figure 2A. In a DCG, each node of the graph represents a residue of which there are two types: those in domain A and those in domain B. An edge joins the two nodes when there is a contact between the residue in domain A and the residue in domain B, with the edge direction being from the node in A to the node in B if a contact exists in conformation 1 (a_1i_→b_1i_) and in the opposite direction if the contact exists in conformation 2 (a_2i_←b_2i_). Figure 2B shows the ‘elemental DCGs’ and the elemental contact changes they represent, namely, maintained, exchanged-partner, exchanged-pair and new. As outlined by Taylor *et al.* (2013), any complex DCG can be decomposed into these elemental DCGs, which allows us to count the number of elemental contact changes involved in the movement. The number of elemental contact changes, N_maint_, N_exchpart_, N_exchpair_ and N_new_ [referred to collectively as **N** where **N** = (N_maint_ N_exchpart_ N_exchpair_ N_new_)], is the primary input for the logistic regression.

### 2.3 Logistic regression

#### Matching domain pairs between DBMM and NRDPDM2d

To perform logistic regression, pairs of structures representing the domain movement in NRDPDM2d need to be matched to pairs of structures in DBMM. NRDPDM is organized by protein family within which the structures are grouped according to a conformational clustering procedure (Qi *et al.*, 2005). We considered there to be a match between a pair of structures in NRDPDM2d and DBMM if both DBMM structures (identified by PDB accession code and chain identifier) are found in the same NRDPDM family.

#### Logistic regression procedure

Let **N**^i^ represent a four-component vector with ${\text{N}}_{\text{1}}^{\text{i}}$ = N_maint,i_, ${\text{N}}_{\text{2}}^{\text{i}}$ = N_exchpart,i_, ${\text{N}}_{\text{3}}^{\text{i}}$ = N_exchpair,i_ and ${\text{N}}_{\text{4}}^{\text{i}}$ = N_new,i_, where N_maint,i_, N_exchpart,i_, N_exchpair,i_ and N_new,i_, denote N_maint_, N_exchpart_, N_exchpair_ and N_new_ in domain movement i, respectively. Let t^i^ = 0 when the DBMM assignment for domain movement i is predominantly hinge, and t^i^ = 1 when the DBMM assignment for domain movement i is predominantly shear. Given labelled training data D = {(**N**^i^,t^i^)}, logistic regression constructs a decision rule that can be used to distinguish between objects belonging to two classes. The logistic regression model is of the form:

(1a) logit(y(N))=w·x+b

where

(1b) $$\text{logit }\left(\text{p}\right)=\log\left(\frac{\text{p}}{\text{1-p}}\right)$$

**w** is a four-component vector of regression coefficients and b is a scalar bias parameter. The optimal value of the regression coefficients is determined by minimizing the cross-entropy training criterion:

(2) $$\text{E}=-\frac{1}{2}\sum_{\text{i}=\text{1}}^{\text{L}}\left[{\text{t}}^{{}_{\text{i}}}\log\left({\text{y}}^{{}_{\text{i}}}\right)+\left(1-{\text{t}}^{{}_{\text{i}}}\right)\log\left({\text{1-y}}^{{}_{\text{i}}}\right)\right]$$

where y^i^ = y(**N**^i^), L is the total number of domain movements in the training set (i.e. the total number of NRDPDM2d domain movements corresponding to the DBMM set).

The output of the logistic regression model can then be regarded as an estimate of the Bayesian a posteriori probability of class membership, i.e.

(3) y(N)≈P(t=1|N)

### 2.4 Translation and Chasles’ theorem

Chasles’ theorem (Chasles, 1830) states that the most general displacement of a rigid body is a screw movement about a unique screw axis. That is, given a rigid body in two different positions (and orientations), the body can be taken from one to the other by a screw movement about a unique screw axis. The DynDom program (Hayward and Berendsen, 1998) determines this screw axis. DynDom produces a PDB-formatted file that contains the structures superposed on one domain together with an ‘arrow molecule’ that depicts the interdomain screw axis. This file allows the calculation of distances between the structures and the interdomain screw axis and can be used for visualizing the domain movement using molecular graphics software. DynDom also gives the rotation angle and translational displacement along the axis that occurs in the screw movement. If the movement is a pure rotation about an axis, then this screw axis is the rotation axis. If a body undergoes a rotation about a structural hinge but also undergoes a translation in the plane of the rotation, then the interdomain screw axis will not coincide with the original hinge axis. Thus, we test for the screw axis being located outside the body of the protein. If this is the case, then we can be sure that there is no control over the rotation being exercised at the axis location, and consequently any rotation about a structural hinge must be accompanied by a translation in the rotation plane. The location of the interdomain screw axis was previously used to define a ‘mechanical hinge’ (Hayward, 1999), it being a bending region (a region of the backbone connecting the two domains within which the rotational transition occurs) with any one of its C^α^-atoms within 5.5 Å of the interdomain screw axis. In proteins not all bending regions are mechanical hinges, but those that are can be thought of as controlling the domain movement much as the hinge of a door helps to determine the location of its rotational axis. An interdomain screw axis that has at least one mechanical hinge has been called an ‘effective hinge axis’ (Hayward, 1999). DynDom also determines the percentage closure. Those with a percentage >50% are annotated here as having a closure motion; those with a percentage ≤50% are annotated as having a twisting motion.

The significance tests made are described in the Supplementary Material.

## 3 RESULTS

### 3.1 Prediction of hinge and shear

Of the 37 ‘predominantly shear’ domain movements in the DBMM, 21 were also in NRDPDM2d, and of the 75 ‘predominantly hinge’ domain movements in the DBMM, 41 were also in NRDPDM2d. To improve statistics, we used the DynDom program directly on structures provided at the DBMM, which gave an extra two examples to add to the 21 from NRDPDM2d in the shear category and an extra 13 to add to the 41 in NRDPDM2d in the hinge category. The training set can be found in the Supplementary Material. The **N**^i^ were calculated for each of the 77 domain movements in the training set, and logistic regression was carried out as described in the Methods section. Logistic regression produced the following model:

(4a) $$\text{y}\left(N\right)=\frac{1}{\text{1}+{\text{e}}^{\alpha }}$$

where

(4b) $$\alpha =-\text{0}{\text{.2387N}}_{\text{maint}}-\text{0}{\text{.0356N}}_{\text{exchpart}}+\text{0}{\text{.4249N}}_{\text{exchpair}}\;+\text{0}{\text{.2122N}}_{\text{new}}+\text{0}\text{.1467}$$

**Fig. 3.**
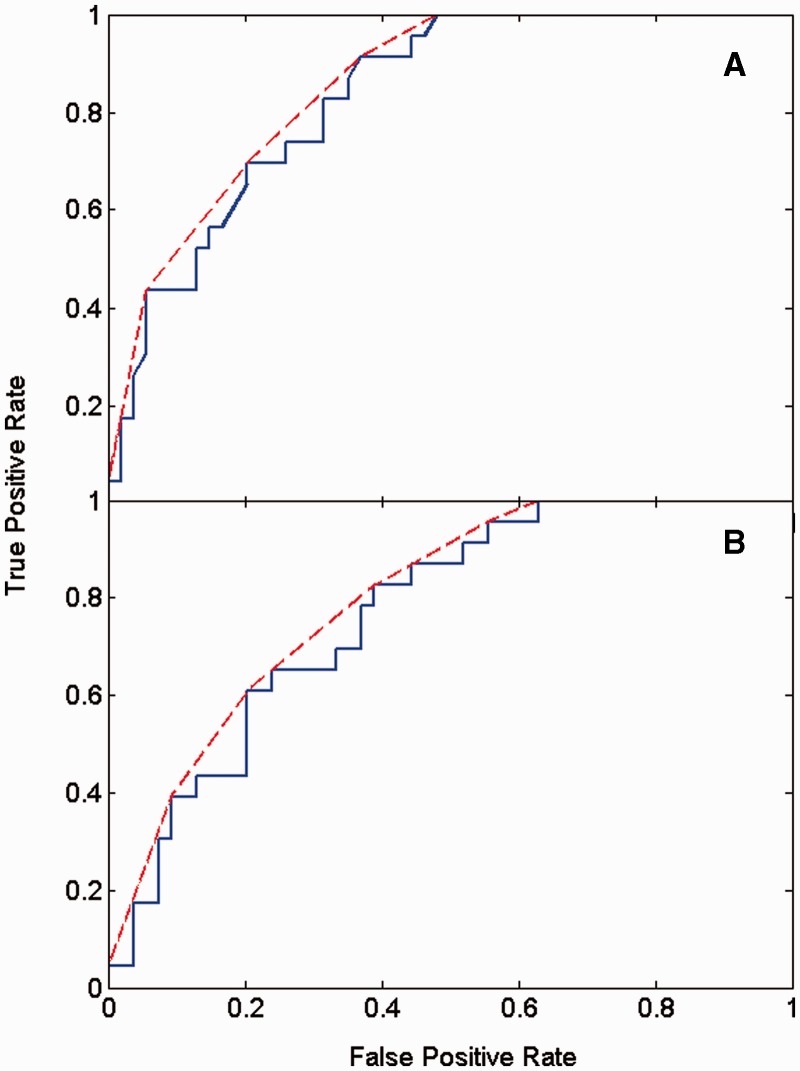
ROC curves for the prediction of hinge and shear using logistic regression. A predictor for shear and hinge was constructed and tested against predominantly shear and predominantly hinge assignments in the DBMM. The ROC curve for the logistic function, given in Equation 4, gives the unbroken line; the convex hull of the unbroken line is the broken line. (A) The area under the ROC curve is 0.83, and the area under the convex hull is 0.86. (B) The ROC curve for a leave-one-out cross-validation approach. The area under the ROC curve is 0.77, and the area under the convex hull, 0.80

To determine whether this model corresponds well to the DBMM assignments, a receiver-operating characteristic curve (ROC) curve was determined. A ROC curve plots the true-positive rate against the false-positive rate. A true positive is a shear correctly predicted shear, and a false positive is a hinge incorrectly predicted shear. The true-positive rate is the number of true positives to number of shear in the dataset, and the false-positive rate is the number of false positives to number of hinge in the dataset. Figure 3A shows the ROC curve. The area under the ROC curve is 0.83, indicating that the logistic function is a good discriminator between hinge and shear movements. To confirm this result, a leave-one-out cross-validation approach was used, the ROC curve of which is shown in Figure 3B. The area under this ROC curve is 0.77, confirming that the logistic function is able to give a good predictor for hinge and shear. Regularized logistic regression (Cessie and Houwelingen, 1992) and kernel logistic regression (Cawley *et al.*, 2007; Cawley and Talbot, 2008) were also tried, but these did not improve on the results obtained using conventional logistic regression.

Before Equation 4 was applied to the NRDPDM2d, the 412 cases where **N** = **0**, were removed, i.e. those cases where N_maint_, N_exchpart_, N_exchpair_ and N_new_ are all equal to zero. The removed movements are those classified as ‘No-contact’, as there are no domain contacts in either conformation. These cases would not be expected to be classed as either shear or hinge according to Gerstein *et al.*, and no such case was found among the 77 DBMM examples. Equation 4 was applied to the remaining 1410 movements in the NRDPDM2d.

**Fig. 4.**
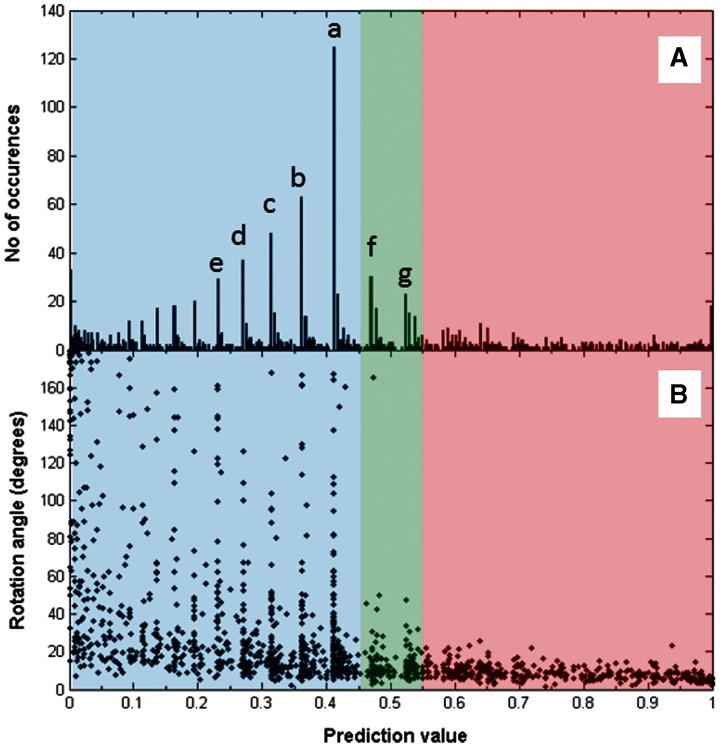
Prediction value distributions. ‘Hinge’, ‘Mixed’ and ‘Shear’ are in the prediction value regions 0.0–0.45, 0.45–0.55 and 0.55–1.0, respectively. (**A**) Histogram of prediction values. The spikes indicated by ‘a’, ‘b’, ‘c’, ‘d’, ‘e’, ‘f’ and ‘g’ correspond to **N** = (0 0 0 1), **N** = (0 0 0 2), **N** = (0 0 0 3), **N** = (0 0 0 4), **N** = (0 0 0 5), **N** = (1 0 0 1) and **N** = (1 0 0 0), respectively. (**B**) The rotation angle plotted against prediction value. The same peaks can be seen and offer an explanation for their existence. For example, the peak at ‘a’ for prediction value 0.411 corresponding to **N** = (0 0 0 1) means there are a large number of domain movements with various angles of rotation that are all able to break a single residue contact pair

Figure 4A shows a histogram for the frequency distribution of the prediction values y. As can be seen, there is no obvious clustering, but there are pronounced peaks at certain values of y. The peaks labelled a,b,c,d,e are due to domain movements where **N** = (0 0 0 N_new_), N_new_ = 1,2,3,4,5, respectively. In our previous work (Taylor *et al.*, 2013), these domain movements are in the ‘Pure new’ class (the most populous after the ‘No-contact’ class), meaning that in one conformation there are no contacts between the domains and in the other conformation there are exactly N_new_ pairwise residue contacts. For these cases, the larger the N_new_, the more ‘hinge-like’ they seem to become in terms of their y value (decreasing with increasing N_new_), although arguments based on the presence or absence of contacts alone might conclude they are all equally domain movements via a hinge mechanism; for all of these, y < 0.45. The peak f, at y = 0.470, is due to the predominance of examples with **N** = (1 0 0 1), which are from the ‘Combined maintained new’ class (the third most populous class). The peak g, at y = 0.523, is from the ‘Pure maintained’ class with **N** = (1 0 0 0) where only one pairwise residue contact is maintained between the domains in the domain movement.

Given that we would like to include all cases in the ‘Pure new’ class as examples of a domain movement via a hinge mechanism, but to be sure that we are excluding weak examples from our classifier, the domain movements were put into three classes as follows:

‘Hinge’, for cases with 0 ≤ y ≤ 0.45; ‘Shear’, for cases with 0.55 ≤ y ≤ 1.0; ‘Mixed’, for cases with 0.45 < y < 0.55.

It is important for the comparisons we intend to make that the two main classes, hinge and shear, have a high precision. The precision of a class can be calculated as the proportion of cases *correctly* predicted to be in that class (true-positive results) to the total number cases predicted to be in that class. Of the 61 DBMM cases predicted hinge, 48 were actually predominantly hinge according to DBMM, giving a precision of 79%. The numbers are low for the calculation of the precision of shear prediction. Only 12 DBMM cases were predicted shear, with 9 of them actually classed as predominantly shear by DBMM, giving a precision of 75%. The natural boundary of 0.5 (so hinge for 0 ≤ y ≤ 0.5 and shear for 0.5 < y ≤ 1.0) lowers the precision for the shear class to below 70%. These results support our choice of 0.45 and 0.55 as the classification boundaries and show that we are able to assign hinge and shear to domain movements automatically with a high degree of correspondence with assignments made using the intuitive method.

Applying the predictor to the 1410 examples, 884 are the hinge class (63%), 361 in the shear class (26%), with the remaining 165 in the mixed class (12%). Out of the whole set of 1822 domain movements, 23% are in the No-contact set, 49% hinge, 20% for shear, and 9% mixed. This means we have a 10-fold increase in the number of examples over the number previously available allowing us to study hinge and shear mechanisms using statistical methods to measure the significance of our results. The result of applying the predicator to the training set can be found in the Supplementary Material.

### 3.2 Rotation angle as indicator of hinge and shear

**Fig. 5.**
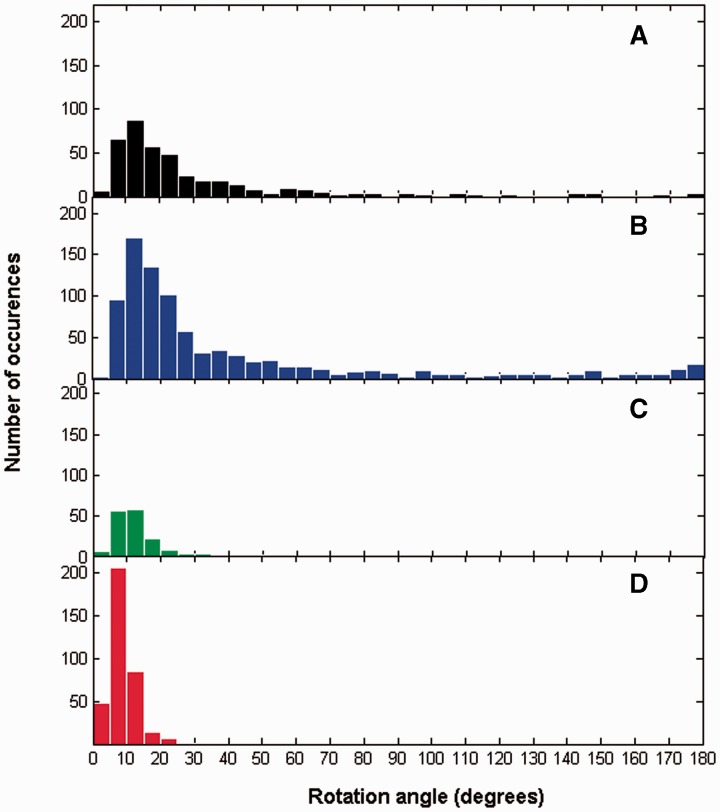
Histograms for rotation angles. (**A**) no-contact set, (**B**) hinge set, (**C**) mixed set, (**D**) shear set

Figure 4B shows the rotation angle plotted against the prediction value. One can discern a general trend for the rotation angle to increase with decreasing prediction value, i.e. the motions become more hinge-like. Large rotations occur below a prediction value of 0.45 in the hinge region. Most of the peaks there correspond to the peaks indicated in Figure 4A and also correspond to the ‘Pure new’ class. In fact, nearly 80% of those peaks in hinge are where N_new_ is larger than N_maint_, N_exchpart_, and N_exchpair_. Figure 5 shows histograms for the rotation angles for the four categories. One can immediately see that for shear, rotations do not exceed 25°. For these cases, there is nearly always either predominance in the number of maintained, N_maint_, or the number of exchanged-partner contact changes, N_exchpart_, indicating that for a preserved-interface movement the angle of rotation is limited to 25°.

Also of interest in Figure 5 is the slight increase in the number of hinge examples where the angle of rotation is close to 180°. Some of these are examples of domain swapping (Bennett *et al.*, 1994).

**Fig. 6.**
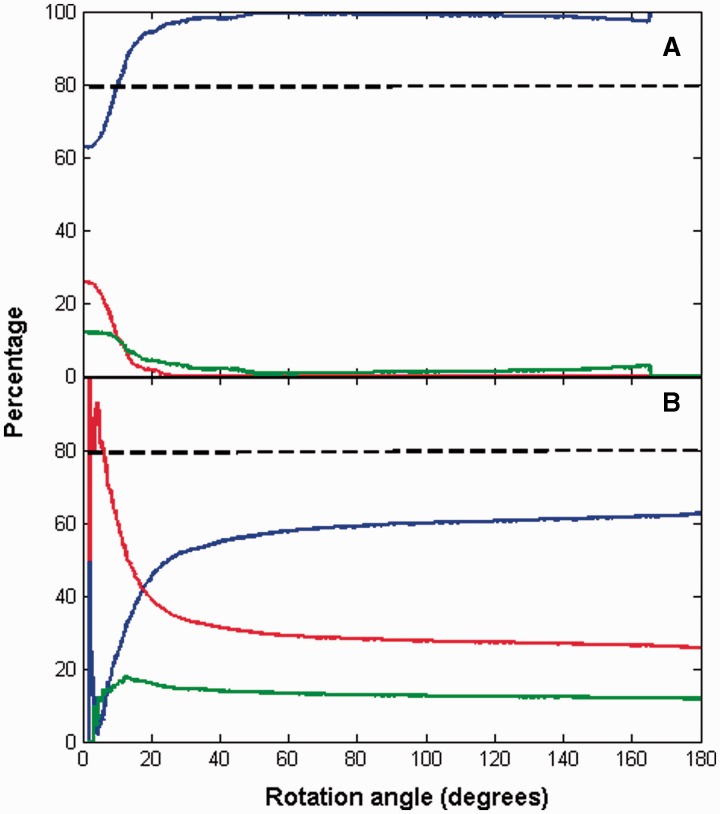
Predictive value of angle of rotation. Blue lines correspond to ‘Hinge’, green lines to ‘Mixed’ and red lines to ‘Shear’. (**A**) A point on a line gives the proportion (in percentage) of domain movements (excluding non-contact cases) with rotation angles greater than or equal to that given at the point, that are from the set indicated by the colour of the line. (**B**) A point on a line gives the proportion (in percentage) of domain movements (excluding non-contact cases) with rotation angles less than that given at the point, that are from the set indicated by the colour of the line

Figures 4 and 5 suggest that the angle of rotation is predictive of whether a domain movement is hinge or shear. Figure 6 shows the extent to which rotation can be used for predicting hinge or shear. In Figure 6A, the blue line gives, among all domain movements (excluding non-contact cases) with rotation angles greater than or equal to any selected threshold value, the proportion that are from the hinge class. It shows that among the set of domain movements (excluding non-contact cases) with rotation angles ≥10°, 80% are hinge. In Figure 6B, the red line gives, among all domain movements (excluding non-contact cases) with rotation angles less than any selected threshold value, the proportion that are from the shear class. It shows that among the set of domain movements (excluding non-contact cases) with rotation angles of <6°, 80% are shear.

### 3.3 Translation in domain movements

If the shear concept relates to translational movement, then one would expect a large proportion of the shear set to have an interdomain screw axis located outside the body of the protein. However, of the 361 shear examples, only five (1.4%) have an axis outside the body of the protein (using a cut-off distance of 5.5 Å between the axis and any heavy atom of the protein). For the 884 hinge examples, 9 (1.0%) have an axis outside the body of the protein. The rarity of axes located outside the body of the protein indicates that translational movements are rare overall. If there is any truth in the concept of shear indicating a translational movement and hinge indicating a rotational movement, then at least one would expect there to be significantly more cases of remote axes in the shear set than the hinge set. Significance testing on this gave a z-value of 0.56, which gives p(z ≥ 0.56) = 29% for the probability that this difference (1.4% versus 1%) or greater occurs by chance. This result suggests that shear movements are just as likely to have a rotational axis within the body of the protein as hinge movements, implying local control, and that shear movements do not involve the relative translation of one domain relative to the other at least without a rotation occurring about an axis within the body of the protein, i.e. translation is in the axis direction. Considering translation in the axis direction, the mean absolute value for the hinge set is 1.47 Å (SD = 3.1 Å), whereas for the shear set the mean is 0.35 Å (SD = 0.37 Å). Thus, there is significantly more translation along the axis in the hinge set than the shear set, but this is likely to be because of the fact that the rotations are larger among the hinge set. Comparing the pitch would make more sense. The mean absolute value of the pitch for the hinge set is 0.043 Å/degree (SD = 0.095 Å/degree), whereas for the shear set the mean is 0.044 Å/degree (SD = 0.058 Å/degree). Again the difference is not significant (*P* = 58%).

We also have tested whether the shear set is significantly more likely not to have an effective hinge axis compared with the hinge set. For shear, 61 examples do not have an effective hinge axis (16.8%), whereas the corresponding value for hinge is 117 (13.2%). With a *P* = 4.7%, this would be significant at the 5% level and suggests that for shear, interactions at the preserved domain interface help control the domain movement, whereas in hinge, it is more likely to be the backbone connections between the domains.

### 3.4 Twisting movements

The presence of exchanged-partner contact changes is a strong indicator for a shear movement. In our previous work, it was argued that when this type of contact change occurs in isolation, then under certain assumptions concerning the shape of the domains and the location of the hinge axis, this is most likely to occur via a ‘sliding twist’ movement. A new contact change or an exchanged-pair contact change would most likely occur via either an open-closed or see-saw domain movement. These movements would be closure movements under the same assumptions. This would suggest that twisting movements are more likely to occur in the shear set than the hinge set. For shear, 114 have a predominantly twisting movement (32.0%), whereas the corresponding value for hinge is 192 (21.7%). With a *P* = 0.012%, this difference is highly significant, showing that twisting movements are more prevalent in the shear set.

### 3.5 Website

We have produced a website (see http://www.cmp.uea.ac.uk/dyndom/interface) where the domain movements are organized according to whether they are in the no-contact, shear (called ‘Interface-preserving movement’, see Discussion section), hinge (called ‘Interface-creating movement’) or mixed set. Each class comprises a list of protein names together with a pair of PDB accession codes and chain identifiers that specify the domain movement. The link provided takes one to a page where the molecular graphics applet, Jmol (http://jmol.sourceforge.net/), is used to display the movement and to indicate the residues that make contact in each conformation. There is also a link to the corresponding DCG classification page and the DynDom page for that domain movement which gives details on the residues comprising the domains, the location of the hinge axis, the hinge-bending residues, the angle of rotation, percentage closure, as well as many other details. A link to the DynDom family page is also provided, which gives a conformational analysis of closely related structures and their domain movements.

## 4 DISCUSSION

The concept of hinge and shear mechanisms in domain movements was introduced nearly 20 years ago. Assignments of domain movements to these mechanisms were made by an intuitive method that is necessarily subjective. This has limited its application to a small number of domain movements. In the past 20 years, the PDB has grown 30-fold in size and with it the number of implied domain movements. The NRDPDM database contains 2035 unique domain movements, and it would be an onerous task to analyse all of these conformational pairs using molecular graphics software, for the purpose of assigning hinge and shear mechanisms. Therefore, an objective, quantitative method that can be implemented computationally for rapid assignment is needed. The difficulty in achieving this lies in the translation of a subjective method to a quantitative method. There are two pieces of information we can use for this purpose: the description of the subjective method used, and the actual assignments themselves. The description suggested that quantities based on the number of instances in each of the four types of residue contact changes from our previous work (Taylor *et al.*, 2013) could be used in distinguishing between preserved interfaces and interface creation. The assignments themselves were used as training data to combine these quantities using logistic regression so as to optimally reproduce the original assignments. The results suggest that we have indeed succeeded in creating a quantitative method for computational assignment of hinge and shear mechanisms. Using this approach, we have managed to classify a much larger set of domain movements into hinge and shear resulting in a 10-fold increase in the number of examples over the number previously available with a high degree of precision.

The term ‘shear’ and the figures used to illustrate the shear mechanism have led many to interpret a domain closure to occur via a relative translation of one domain relative to the other. Although this is possible, our results have shown that this is rare overall, and no more likely to occur among the shear set than the hinge set. We suggest that the term ‘shear movement’ is better referred to as ‘interface-preserving movement’ and ‘hinge’ as ‘interface-creating movement’. These more prosaic terms are still broadly consistent with the original concept but should not lead to misinterpretation.

Our analysis has shown that for proteins with domain movements classified as shear, the movement does not involve a significant translation of the two domains but a rotation about an axis within the body of the protein just as for a protein undergoing a domain movement via the hinge mechanism. We have shown that maintained and exchanged-partner contact changes are strong indicators for shear, whereas exchanged-pair and new contact changes are strong indicators for hinge. The finding that there are significantly more twisting movements in the shear set than in the hinge set is consistent with the notion that a twisting movement can preserve the domain interface. This offers one explanation of how a rotational movement can preserve an interface without relative translation. However, not all predominantly interface-preserving movements occur via a twisting motion; many can still occur via a closure motion by rotation about well-defined hinges.

The case of citrate synthase illustrates how a ‘predominantly shear’ movement as designated by DBMM would still be appropriately described as hinge-bending even though it is in our mixed class (prediction value of 0.55) with slightly more interface-preserving features than interface creating. Figure 2A shows the DCG for citrate synthase. There are 10 maintained contact changes, 2 exchanged-partner contact changes, 2 exchanged-partner contact changes and 6 new contact changes. It has a well-defined hinge axis created by mechanical hinges, one of which is a ‘hinged-loop’ (Hayward, 1999), a loop flanked by two bending regions through which the hinge axis passes. This hinged-loop clearly helps control the domain movement just as a hinge would in a protein conventionally regarded as undergoing closure via hinge bending, e.g. lactoferrin. The domain movement in citrate synthase is also an example of a protein that undergoes closure (84%) via hinge bending, but one that preserves some part of the domain interface.

## Supplementary Material

Supplementary Data
