# Supplementary material for: Quantitative method for the assignment of hinge and shear mechanism in protein domain movements
Source: Bioinformatics. 2014 Jul 30;30(22):3189–96. doi: 10.1093/bioinformatics/btu506 (PMC4221117; doi:10.1093/bioinformatics/btu506)
Supplement: Supplementary Data [file supp_30_22_3189__index.html]

Quantitative Method for the Assignment of Hinge and Shear Mechanism in Protein Domain Movements — Quantitative method for the assignment of hinge and shear mechanism in protein domain movements — Quantitative method for the assignment of hinge and shear mechanism in protein domain movements — Supplementary Data 

# Quantitative method for the assignment of hinge and shear mechanism in protein domain movements

## Supplementary Data

files

**Files in this Data Supplement:**

- Supplementary Data - docx file
