# Supplementary material for: Quantitative method for the assignment of hinge and shear mechanism in protein domain movements
Source: Bioinformatics. 2014 Jul 30;30(22):3189–96. doi: 10.1093/bioinformatics/btu506 (PMC4221117; doi:10.1093/bioinformatics/btu506)
Supplement: Supplementary Data [file supp_btu506_Supplementary_Material.docx]

**Training Set for Logistic Regression**

| **Protein Name** | **DBMM Assignment** | **PDB 1** | **ChainID1** | **PDB 2** | **Chain ID2** | **Rotation Angle (deg)** | **Prediction value** | **Predicted Class** |
| --- | --- | --- | --- | --- | --- | --- | --- | --- |
| Alcohol Dehydrogenase (ADH) | Predominantly Shear | 1N8K | A | 1YE3 | A | 8.5 | 0.171 | Hinge |
| Aspartate Amino Transferase (AAT) | Predominantly Shear | 1AKB | A | 7AAT | B | 13.3 | 0.314 | Hinge |
| Calpain protease core | Predominantly Shear | 2G8J | A | 1TL9 | A | 13.4 | 0.319 | Hinge |
| Citrate Synthase | Predominantly Shear | 1CTS | null | 1CSH | null | 19.4 | 0.547 | Mixed |
| Glyceraldehyde-3-phosphate Dehydrogenase | Predominantly Shear | 2GD1 | R | 1NQ5 | A | 8.3 | 0.443 | Hinge |
| SARS virus protease | Predominantly Shear | 1Z1J | A | 1UK2 | A | 13.9 | 0.563 | Shear |
| Trp Repressor (TrpR) | Predominantly Shear | 1ZT9 | D | 1WRP | R | 12.1 | 0.647 | Shear |
| Actin | Predominantly Shear | 1HLU | A | 1MDU | E | 13.5 | 0.164 | Hinge |
| Aspartyl tRNA Synthetase | Predominantly Shear | 1G51 | B | 1EFW | A | 11.4 | 0.426 | Hinge |
| Cytochrome P450BM-3 | Predominantly Shear | 1JPZ | B | 1BVY | A | 15.1 | 0.759 | Shear |
| DNA Polymerase III | Predominantly Shear | 1MMI | A | 1JQL | A | 13.7 | 0.375 | Hinge |
| E. coli clamp loader gamma subunit | Predominantly Shear | 1JR3 | D | 1XXH | A | 18.3 | 0.591 | Shear |
| E. Coli Mta/Adohcy Nucleosidase | Predominantly Shear | 1NC1 | A | 1JYS | B | 8.6 | 0.622 | Shear |
| Endothiapepsin | Predominantly Shear | 1GVU | A | 4APE | null | 3.8 | 0.999 | Shear |
| Glutamyl tRNA synthetase | Predominantly Shear | 1N78 | B | 1GLN | null | 10.9 | 0.670 | Shear |
| Heat Shock Protein (HSP) | Predominantly Shear | 1HX1 | A | 1KAZ | null | 12.7 | 0.265 | Hinge |
| Hexokinase | Predominantly Shear | 2YHX | A | 1HKG | A | 13 | 0.789 | Shear |
| Molybdate-binding protein | Predominately Shear | 1HK9 | A | 1H9M | A | 8.5 | 0.311 | Hinge |
| Phenylalanine Hydroxylase | Predominantly Shear | 1MMK | A | 1J8U | A | 16.1 | 0.270 | Hinge |
| Phosphofructokinase (PFK) (not allosteric transition) | Predominantly Shear | 1PFK | A | 1PFK | B | 5.3 | 0.864 | Shear |
| PvuII endonuclease | Predominantly Shear | 1NI0 | B | 3PVI | A | 33.3 | 0.411 | Hinge |
| Threonine tRNA Synthetase | Predominantly Shear | 1EVK | A | 1EVL | D | 11.4 | 0.441 | Hinge |
| Tyrosine Kinase-Type Cell Surface Receptor Her2 | Predominantly Shear | 1N8Z | B | 2FJG | B | 20.3 | 0.411 | Hinge |
| ATP Sulfurylase | Predominantly Hinge | 1I2D | C | 1M8P | A | 21 | 0.167 | Hinge |
| cAMP-dependent Protein Kinase (catalytic domain) | Predominantly Hinge | 1JLU | E | 1CMK | E | 13.4 | 0.361 | Hinge |
| c-Src tyrosine kinase | Predominantly Hinge | 1YI6 | B | 1FMK | A | 22.1 | 0.314 | Hinge |
| Folylpolyglutamate Synthetase | Predominantly Hinge | 1JBW | A | 2GC5 | A | 15.4 | 0.132 | Hinge |
| HCV Helicase | Predominantly Hinge | 8OHM | null | 1CU1 | B | 35.3 | 0.063 | Hinge |
| T7 Phage RNA Polymerase | Predominantly Hinge | 1ARO | P | 1CEZ | A | 11.8 | 0.751 | Shear |
| Thioredoxin reductase/Glutathione reductase | Predominantly Hinge | 1F6M | B | 1TRB | null | 65.8 | 0.212 | Hinge |
| Transferrins (N-terminal lobe) | Predominantly Hinge | 1RYO | A | 1BP5 | C | 62.9 | 0.003 | Hinge |
| Troponin-C | Predominantly Hinge | 1YTZ | C | 1TOP | null | 47.8 | 0.029 | Hinge |
| Uracil-DNA Glycosylase | Predominantly Hinge | 1EMH | A | 2HXM | A | 6.7 | 0.895 | Shear |
| 3-Isopropylmalate Dehydrogenase | Predominantly Hinge | 1OSJ | A | 1IDM | null | 15.3 | 0.253 | Hinge |
| Acetylcholinesterase | Predominantly Hinge | 2CMF | A | 2J4F | A | 5.2 | 0.525 | Mixed |
| Acetyl-CoA synthase | Predominantly Hinge | 1OAO | D | 1MJG | N | 52.1 | 0.010 | Hinge |
| Adenylate Kinase (ADK) | Predominantly Hinge | 1E4V | A | 1E4Y | B | 15.7 | 0.270 | Hinge |
| Arabinose, Leucine, and Galactose Binding Proteins | Predominantly Hinge | 2FW0 | A | 2HPH | A | 35.4 | 0.137 | Hinge |
| Biotin carboxylase | Predominantly Hinge | 1BNC | A | 1DV2 | A | 47.1 | 0.195 | Hinge |
| C. Glutamicum DAP Dehydrogenase | Predominantly Hinge | 1F06 | B | 2DAP | null | 10.7 | 0.079 | Hinge |
| Calmodulin | Predominantly Hinge | 1QX5 | J | 1QX7 | R | 13.7 | 0.282 | Hinge |
| Catabolite Gene Activator Protein (CAP) | Predominantly Hinge | 1ZRE | B | 1O3Q | A | 12 | 0.314 | Hinge |
| CBL | Predominantly Hinge | 1B47 | A | 1YVH | A | 16.1 | 0.319 | Hinge |
| Cell Adhesion Molecule CD2 | Predominantly Hinge | 1CDC | A | 1A64 | A | 97.7 | 0.113 | Hinge |
| Cyanovirin-N | Predominantly Hinge | 1L5B | B | 3EZM | A | 72 | 0.411 | Hinge |
| Diphtheria Toxin (DT) | Predominantly Hinge | 1F0L | B | 1TOX | B | 177.4 | 0.000 | Hinge |
| DNA Beta-Glucosyltransferase | Predominantly Hinge | 1JEJ | A | 1M5R | A | 14.8 | 0.162 | Hinge |
| DNA Polymerase Beta (Pol Beta) | Predominantly Hinge | 2FMP | A | 7ICO | A | 37.1 | 0.314 | Hinge |
| E. coli. Periplasmic Dipeptide Binding Protein | Predominantly Hinge | 1DPE | null | 1DPP | A | 53.7 | 0.001 | Hinge |
| Elongation Factor G | Predominantly Hinge | 2EFG | A | 1FNM | A | 13.2 | 0.418 | Hinge |
| Eukaryotic RNA Polymerase | Predominantly Hinge | 1I50 | A | 1I6H | A | 30.1 | 0.223 | Hinge |
| Ferric binding protein | Predominantly Hinge | 1MRP | null | 1NNF | A | 21.9 | 0.153 | Hinge |
| Formate Dehydrogenase (FDH) | Predominantly Hinge | 2NAC | A | 2NAD | A | 8.1 | 0.265 | Hinge |
| Glur2 ligand-binding core | Predominantly Hinge | 2I3V | A | 2CMO | A | 26 | 0.113 | Hinge |
| Glutamate Dehydrogenase | Predominantly Hinge | 1AUP | null | 1HRD | C | 24.7 | 0.015 | Hinge |
| Glutamine Binding Protein | Predominantly Hinge | 1GGG | A | 1WDN | A | 55.7 | 0.052 | Hinge |
| Glutaminyl-tRNA synthase | Predominantly Hinge | 1GTR | A | 1NYL | A | 9.6 | 0.470 | Mixed |
| Glycerate Dehydrogenase (GDH) | Predominantly Hinge | 1PSD | A | 1YBA | D | 12.5 | 0.470 | Mixed |
| GroEL domain | Predominantly Hinge | 1AON | G | 1XCK | N | 84.5 | 0.164 | Hinge |
| Guanylate Kinase | Predominantly Hinge | 1EX6 | B | 1EX7 | A | 47 | 0.361 | Hinge |
| Kinesin-like KIF1A Motor Domain | Predominantly Hinge | 1I5S | A | 1VFV | A | 23.1 | 0.622 | Shear |
| Lactoferrin | Predominantly Hinge | 1CB6 | A | 1LCF | null | 55.2 | 0.015 | Hinge |
| Lysine/Arginine/Ornithine (LAO) binding protein | Predominantly Hinge | 2LAO | null | 1LST | n | 51 | 0.063 | Hinge |
| Maltodextrin Binding Protein (MBP) | Predominantly Hinge | 1OMP | A | 3MBP | A | 34.8 | 0.034 | Hinge |
| Methylene-Tetrahydromethanopterin Dehydrogen | Predominantly Hinge | 1LU9 | A | 1LUA | A | 8.3 | 0.536 | Mixed |
| mRNA capping enzyme | Predominantly Hinge | 1CKO | A | 1CKM | B | 31.9 | 0.137 | Hinge |
| Mura (Udp-N-Acetylglucosamine Enolpyruvyltransferase | Predominantly Hinge | 1UAE | null | 1EJD | B | 17.2 | 0.020 | Hinge |
| Oligopeptide-binding protein | Predominantly Hinge | 1RKM | null | 1JET | A | 25.7 | 0.008 | Hinge |
| Phosphate-binding protein | Predominantly Hinge | 1QUK | A | 1OIB | A | 26.2 | 0.094 | Hinge |
| Phosphoglycerate Kinase | Predominantly Hinge | 13PK | A | 1PHP | A | 27.1 | 0.025 | Hinge |
| Replication protein A DNA-binding domain | Predominantly Hinge | 1FGU | B | 1JMC | A | 96.2 | 0.019 | Hinge |
| Ribose Binding Protein | Predominantly Hinge | 1BA2 | A | 2DRI | null | 62.9 | 0.028 | Hinge |
| Ribose-5-Phosphate Isomerase | Predominantly Hinge | 1KS2 | A | 1KS2 | B | 12.1 | 0.439 | Hinge |
| T4 lysozyme mutants: Ile3->Pro & Met6->Ile | Predominantly Hinge | 1L96 | A | 1L97 | A | 31 | 0.411 | Hinge |
| Tryptophan Synthase | Predominantly Hinge | 2TYS | B | 1QOQ | B | 14.9 | 0.012 | Hinge |
| Type-C Inorganic Pyrophosphatase | Predominantly Hinge | 1K20 | B | 1WPP | A | 19.2 | 0.023 | Hinge |
| Various Kinases (Tyr, Ser, Thr) | Predominantly Hinge | 2FYS | A | 2OJG | A | 48.1 | 0.124 | Hinge |

**Significance Testing**

Our data are divided into two main sets, Shear and Hinge, between which we are testing whether there is a significant difference in the values associated with a particular feature. Given our sets are large a Normal approximation to the Binomial is made. Let N_H_,${\bar{\text{x}}}_{\text{H}}$, and $\text{σ}_{\text{H}}\text{ }$denote the number examples, the mean value of a particular feature and its standard deviation, respectively, in the Hinge set and N_S_, ${\bar{\text{x}}}_{\text{S}}$, and $\text{σ}_{\text{S}}$the equivalent quantities for the Shear set. The z-value is calculated as:

$\text{z=}\frac{{\bar{\text{x}}}_{\text{S}} \text{- }{\bar{\text{x}}}_{\text{HS}}}{\sqrt{\frac{\text{σ}_{\text{S}}^{\text{2}}}{\text{N}_{\text{S}}\text{ }}\text{ + }\frac{\text{σ}_{\text{H}}^{\text{2}}}{\text{N}_{\text{H}}}}}$ (1)

In the case where we count the number of examples in a set possessing a particular feature of interest we use the following test. Let n_H_ be the number of examples in the Hinge set that possess the feature and n_S_ be the number in the Shear set that possess the feature. Under the null hypothesis of there being no difference between the two sets with respect to this feature, the probability of its occurrence is:

$\text{p=}\frac{\text{n}_{\text{H}}\text{+}\text{n}_{\text{S}}}{\text{N}_{\text{H}}\text{+}\text{N}_{\text{S}}}$ (2)

and the z-value for the difference in the proportions amongst the two sets for this feature would be given by:

$\text{z=}\frac{\frac{\text{n}_{\text{S}}}{\text{N}_{\text{S}}} \text{- }\frac{\text{n}_{\text{H}}}{\text{N}_{\text{H}}}}{\sqrt{\text{pq}\left( \frac{\text{1}}{\text{N}_{\text{H}}}\text{ + }\frac{\text{1}}{\text{N}_{\text{S}}} \right)}}$ (3)

where q=1-p.
